# Predilection of Low Protein C-induced Spontaneous Atherothrombosis for the Right Coronary Sinus in Apolipoprotein E deficient mice

**DOI:** 10.1038/s41598-018-32584-y

**Published:** 2018-10-10

**Authors:** Marco Heestermans, Amber B. Ouweneel, Jasmin Hassan, Meander Kloosterman, Pieter H. Reitsma, Marion J. J. Gijbels, Bart J. M. van Vlijmen, Miranda van Eck

**Affiliations:** 1 Einthoven Laboratory for Vascular and Regenerative Medicine, Leiden University Medical Center, Leiden, The Netherlands; 2 Department of Internal Medicine, Division of Thrombosis and Hemostasis, Leiden University Medical Center, Leiden, The Netherlands; 3 Division of BioTherapeutics, Leiden Academic Centre for Drug Research, Leiden University, Leiden, The Netherlands; 4 Department of Pathology and department of Molecular Genetics, Cardiovascular Research Institute Maastricht, Maastricht, The Netherlands; 5 Department of Medical Biochemistry, Amsterdam Medical Center, Amsterdam, The Netherlands

## Abstract

Silencing of anticoagulant protein C using RNA interference (si*Proc*) evokes low incident but spontaneous atherothrombosis in the aortic root of apolipoprotein E–deficient (*Apoe*^−/−^) mice. The aims of the current study were (1) to analyze if plaque characteristics or circulating factors could be linked to atherothrombosis susceptibility, (2) to increase the incidence of atherothrombosis by transiently increasing blood pressure, and (3) to direct atherothrombosis to an additional predefined vascular site by applying a semi-constrictive collar around the carotid artery. si*Proc*-driven spontaneous atherothrombosis in the aortic root of *Apoe*^−/−^ mice was reproduced and occurred at an incidence of 23% (9 out of 39 mice), while the incidence of collar-induced atherothrombosis in the carotid artery was 2.6% (1 out of 39 mice). Treatment with phenylephrine, to transiently increase blood pressure, did not increase atherothrombosis in the aortic root of the *Apoe*^−/−^ mice nor in the carotid arteries with collars. Plaques in the aortic root with an associated thrombus were lower in collagen and macrophage content, and mice with atherothrombosis had significantly more circulating platelets. Plasma protein C, white blood cell counts, total cholesterol, fibrinogen, serum amyloid A, and IL-6 were not different amongst si*Proc* treated mice with or without thrombosis. Remarkably, our data revealed that thrombus formation preferably occurred on plaques in the right coronary sinus of the aortic root. In conclusion, there is a predilection of low protein C-induced spontaneous atherothrombosis in *Apoe*^−/−^ mice for the right coronary sinus, a process that is associated with an increase in platelets and plaques lower in collagen and macrophage content.

## Introduction

Atherothrombosis, characterized by superimposed thrombus formation overlying a ruptured or eroded atherosclerotic lesion, is the cause of death for more than 14 million individuals per year worldwide (in 2015, World Health organization, www.who.int). Atherogenesis, which can eventually lead to atherothrombosis, has been studied extensively in genetically modified mouse models: When mice are deficient for genes involved in cholesterol metabolism, such as in apolipoprotein E knockout (*Apoe*^−/−^) mice or low-density lipoprotein receptor knockout (*Ldlr*^−/−^) mice, are fed a cholesterol-rich diet, they rapidly develop atherosclerotic plaques^1–3^. These mouse models have proven to be valuable for unraveling the pathophysiology of atherosclerosis^4,5^. However, unlike in humans, atherothrombosis does not occur spontaneously in mice, although some signs of rupture or erosion and intraplaque hemorrhage have been recorded^6–8^.

Recently, we showed that transient (7 days) siRNA mediated lowering of the natural anticoagulant protein C (si*Proc*) in atherosclerotic *Apoe*^−/−^ mice induced superimposed thrombus formation on atherosclerotic plaques in the aortic root^9^. Although the incidence of this unique phenotype was low (1 out of 4 and 3 out of 25 in two independent experiments, cumulative incidence of 14%), our novel mouse model might be of use to better understand the pathophysiology of atherothrombosis. Moreover, a mouse model of atherothrombosis will be instrumental for the development of novel strategies for the treatment and prevention of atherothrombosis in humans in the future.

Currently, the factors which determine when and where a thrombus develops on top of an atherosclerotic plaque are still largely unknown. We hypothesized that the incidence of atherothrombosis may increase when an additional risk factor for atherothrombosis is introduced on top of the impaired anticoagulant activity by knockdown of protein C. An important risk factor for the development of atherothrombosis and consequent cardiovascular hospitalization is pulse pressure^10^. A transient increase in blood pressure can be achieved in animal models by the administration of phenylephrine (PE), a selective α1-adrenergic receptor agonist. This will increase the strain on atherosclerotic plaques and thereby stimulate the risk of plaque rupture/erosion and subsequent atherothrombosis. In line, PE administration was associated with increased rupture of p53 treated plaques in *Apoe*^−/−^ mice^11^. In the current study we therefore also applied PE treatment to increase the strain on the atherosclerotic plaques and stimulate atherothrombosis incidence in the si*Proc* treated *Apoe*^−/−^ mice. Moreover, we attempted to direct atherothrombosis to the carotid artery by placing perivascular collars around the carotid artery, a procedure which induces rapid atherogenesis proximal of the collars^12^. This allowed us to investigate the impact of low protein C on plaques at an additional predefined vascular site and of a different origin than the plaques in the aortic root.

## Results

**Figure 1:**
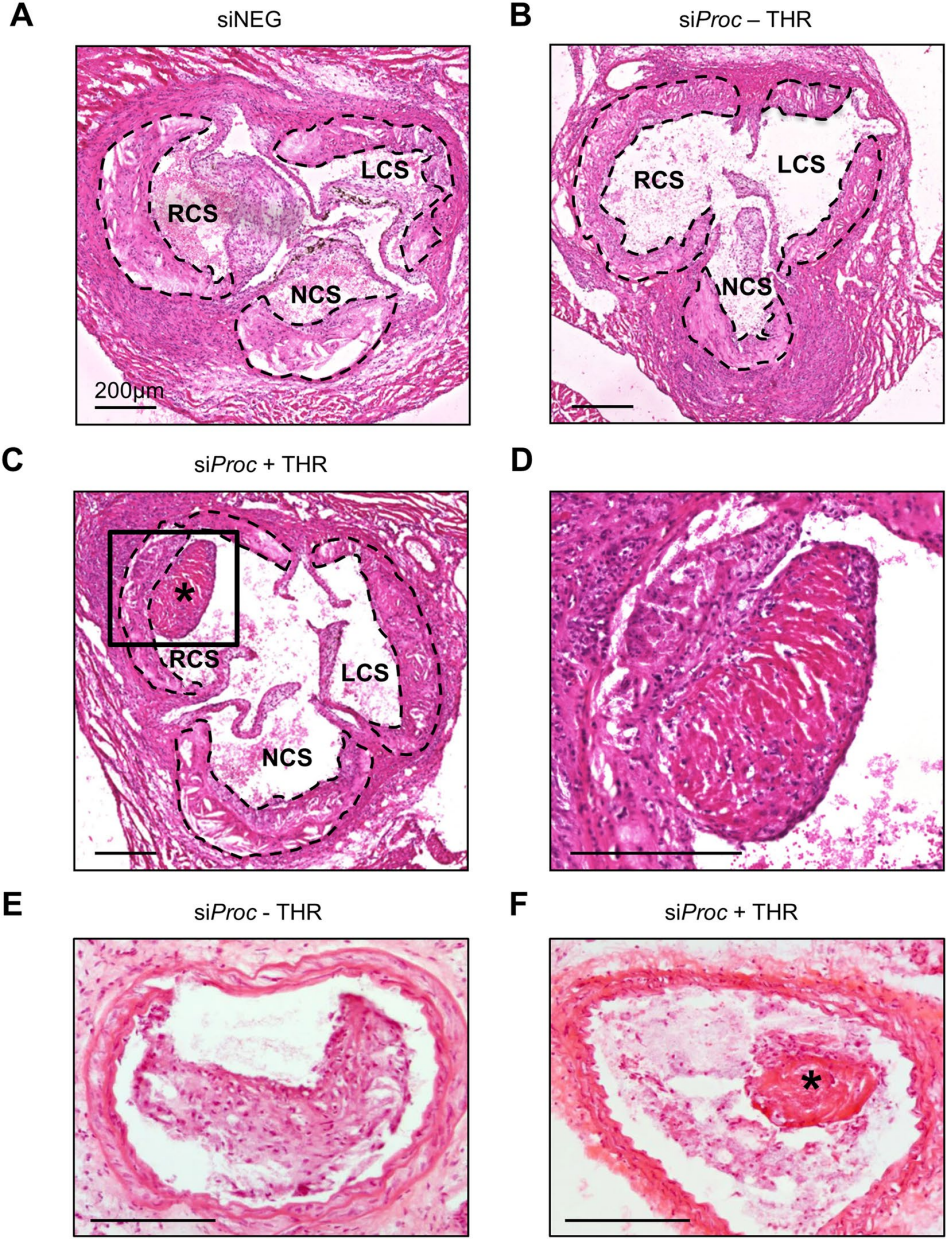
Atherothrombosis in the aortic root of si*Proc* treated *Apoe*^−/−^ mice. (**A**) Representative section of the aortic root of a mouse treated with siNEG. (**B**) Representative section of the aortic root of a mouse treated with si*Proc*, without a thrombus (si*Proc* − THR). (**C** and **D**) Representative section of the aortic root of a mouse treated with si*Proc*, with a thrombus associated with an atherosclerotic plaque (si*Proc* + THR, panel D is a magnification of the black square in panel C). (**E**) Representative section of the common carotid artery containing an atherosclerotic lesion upon collar placement, derived from an si*Proc* treated mouse. (**F**) A thrombus-like structure associated with an atherosclerotic lesion in the common carotid artery. All sections are hematoxylin and eosin (HE) stained. The scattered black lines in panels A, B, and C mark borders of the plaque. LCS: Left coronary sinus, NCS: Non-coronary sinus, RCS: Right coronary sinus. *Thrombus. Black bars represent 200 μm.

**Figure 2:**
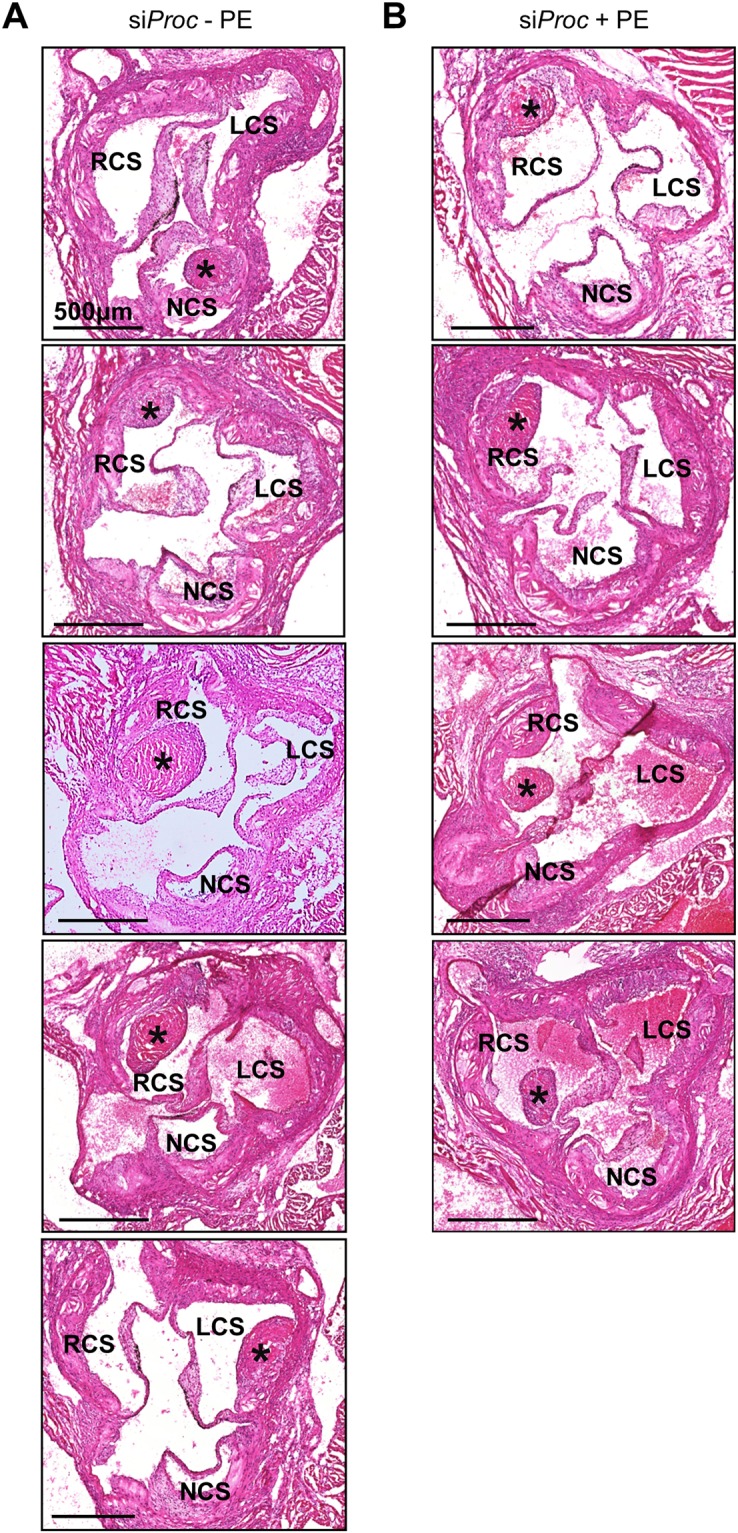
Overview of all si*Proc* associated atherothrombotic events in the aortic root of *Apoe*^−/−^ mice. (**A**) In 5 mice in the si*Proc* group without phenylephrine (si*Proc* − PE) treatment atherothrombosis in the aortic root was observed. (**B**) In 4 mice in the si*Proc* group with phenylephrine (si*Proc* + PE) treatment atherothrombosis in the aortic root was observed. All sections were HE stained. Black bars represent 500 μm. LCS: Left coronary sinus, NCS: Non-coronary sinus, RCS: Right coronary sinus. *Thrombus.

At the time of sacrifice, all *Apoe*^−/−^ mice treated with PBS or phenylephrine (PE) and siRNA (both siNEG and si*Proc*) appeared healthy and did not show any abnormalities. Histological analysis of the aortic root of 10 weeks WTD-fed si*Proc* treated mice demonstrated the presence of atherosclerotic plaque-associated thrombi, with a similar size and composition as previously reported^9^. In total, 9 out of 39 si*Proc* treated *Apoe*^−/−^ mice (23.0%, CI (95%): 16.7–47.9) were categorized as atherothrombosis-positive in the aortic root (si*Proc* + THR, Figs 1A–D and 2). In addition to the 9 si*Proc* + THR mice with typical plaque-associated thrombi, 2 mice developed atypical clots in the aortic root. One atypical clot, although fibrin-positive and clearly associated with an atherosclerotic plaque, did not have the layered structure and did not contain leukocytes as the typical thrombi. These are histological arguments for being a fresh thrombus (Supplemental Fig. 1A). The second atypical thrombus was associated with a valve, although present in the aortic sinus. Despite its unusual location, the thrombus was similar to the typical thrombi found on top of the atherosclerotic plaques. The valve with which it was associated contained a high number of leukocytes, but this was not unique for this specific heart valve (Supplemental Fig. 1B). Mice with atypical thrombi were included in the si*Proc* mice without a thrombus (si*Proc* − THR) during further analyses.

si*Proc* mice treated with PE had a similar incidence of atherothrombosis compared to PBS treated si*Proc* mice (4 out of 19 vs. 5 out of 20 mice, respectively, Fig. 2).

To investigate if an additional predefined site allowing atherothrombosis development could be created, perivascular collars were placed around both the carotid arteries of the *Apoe*^−/−^ mice, 4 weeks prior to siRNA treatment. In line with previous studies^12,13^, histological analysis of the carotid arteries showed that collar placement resulted in atherosclerotic plaque formation proximal to the collar. Although gross visual inspection revealed a large variation in plaque size and composition within groups, these were not different at the site of maximal stenosis between mice in the different groups (Supplemental Fig. 2). In total, 1 out of 39 si*Proc* mice presented a structure identified as a thrombus in one of the carotid arteries (Fig. 1E,F). The thrombus was associated with the plaque, was rich in erythrocytes, and had a layered structure, and it was comparable to the thrombi found in the aortic root. siNEG treated mice did not show events or structures identified as a thrombus, neither in the aortic root and nor in the carotid arteries. It should however be noted that in our long term experience with collar placement around the carotid arteries of *Apoe*^−/−^ mice, the occurrence of thrombosis as a complication of the procedure is not a rare event. Hence, atherothrombosis induced by silencing of *Proc* seems to be restricted to the aortic root.

**Figure 3:**
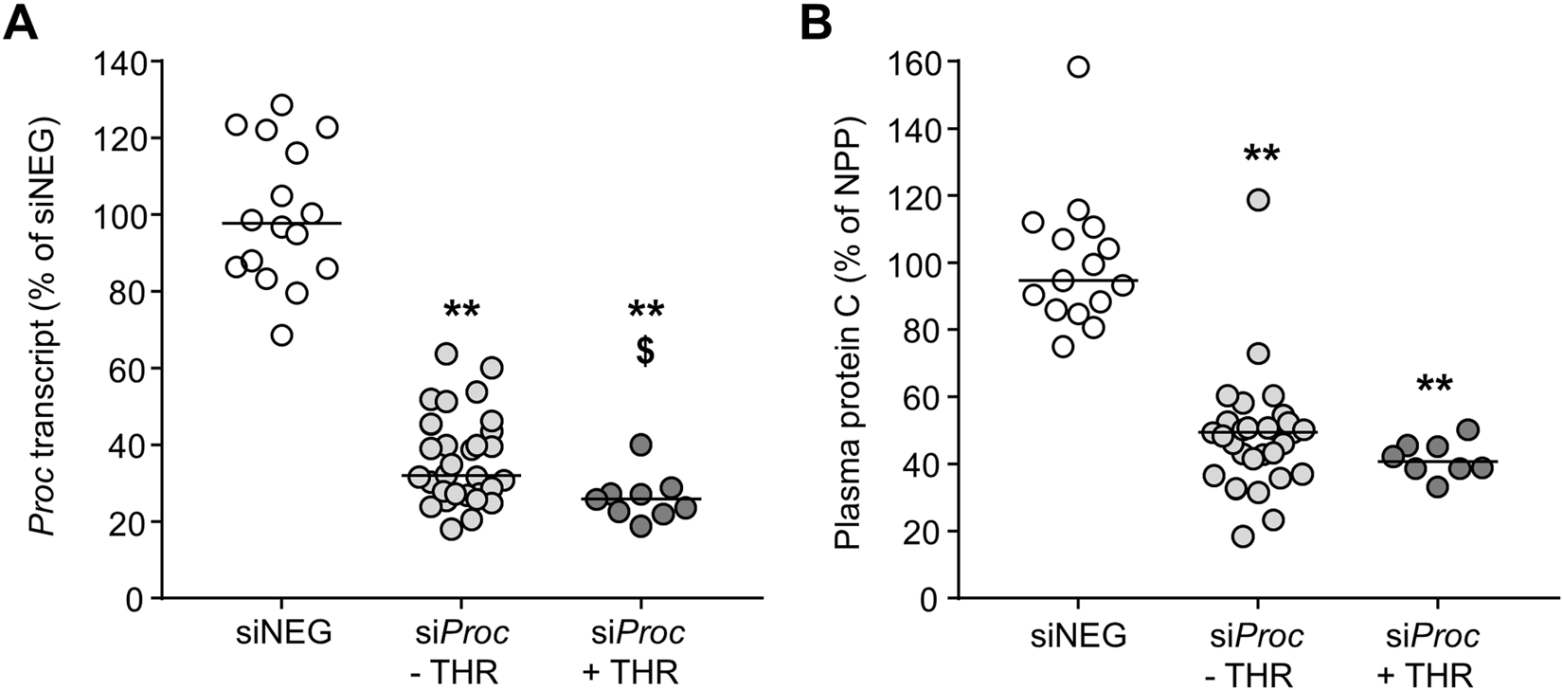
*Proc* liver transcript and plasma protein C levels of siNEG and si*Proc* treated *Apoe*^−/−^ mice. (**A**) *Proc* transcript in the liver upon sacrifice (7 days after si*Proc* treatment) compared to the mean value of siNEG treated mice (100%). (**B**) Plasma protein C levels, measured by ELISA, compared to the mean value of siNEG treated mice (100%). Black bars indicate the median. **P < 0.01 for siNEG vs. si*Proc*. ^$^*P* < 0.05 for si*Proc* − THR vs. si*Proc* + THR.

Livers and blood/plasma were analyzed to determine whether specific markers could be correlated to atherothrombosis susceptibility. As expected, si*Proc* treatment strongly decreased hepatic *Proc* transcript levels, compared to siNEG treated mice (remaining *Proc* transcript: si*Proc* − THR: 32.0% (18.0–63.7), si*Proc* + THR: 25.8% (18.8–40.0), *P* < 0.001, Fig. 3A as compared to siNEG treated mice). Interestingly, the *Proc* transcript in si*Proc* treated mice was significantly lower in si*Proc* + THR, compared to the si*Proc* − THR mice (*P* = 0.013, Fig. 3A). Protein C plasma levels were also reduced in si*Proc* treated mice, as compared to the siNEG treated mice (remaining plasma protein C: si*Proc* − THR: 49.4% (18.4–118.7), si*Proc* + THR: 40.5% (33.2–50.3), *P* < 0.001, Fig. 3B as compared to siNEG treated mice). However, in contrast to the *Proc* transcript data, protein C plasma levels did not reach statistical significance between si*Proc* − THR and si*Proc* + THR mice (*P* = 0.11, Fig. 3B). No differences were observed in mice treated with PE in both the siNEG or siProc group (Supplemental Fig. 3).

**Figure 4:**
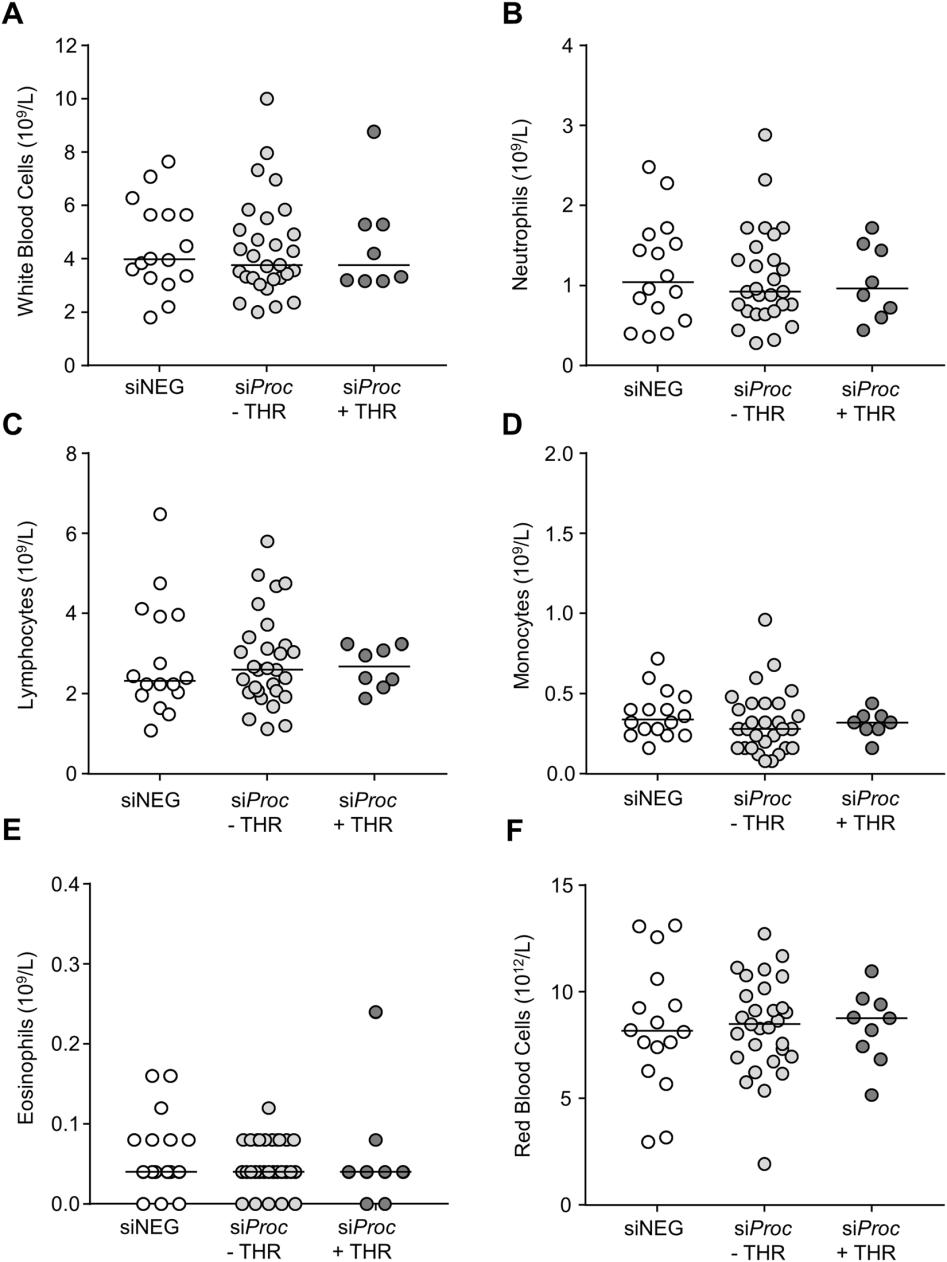
Blood cell populations of siNEG and si*Proc* treated *Apoe*^−/−^ mice. Blood cell numbers from the si*Proc* treated group is divided in plaques without a thrombus (si*Proc* − THR) and plaques containing a thrombus (si*Proc* + THR). (**A**) Total white blood cells, (**B**) neutrophils, (**C**) lymphocytes, (**D**) monocytes, (**E**) eosinophils, and (**F**) red blood cells are displayed. Black bars indicate the median. No significant differences between the groups were scored.

**Figure 5:**
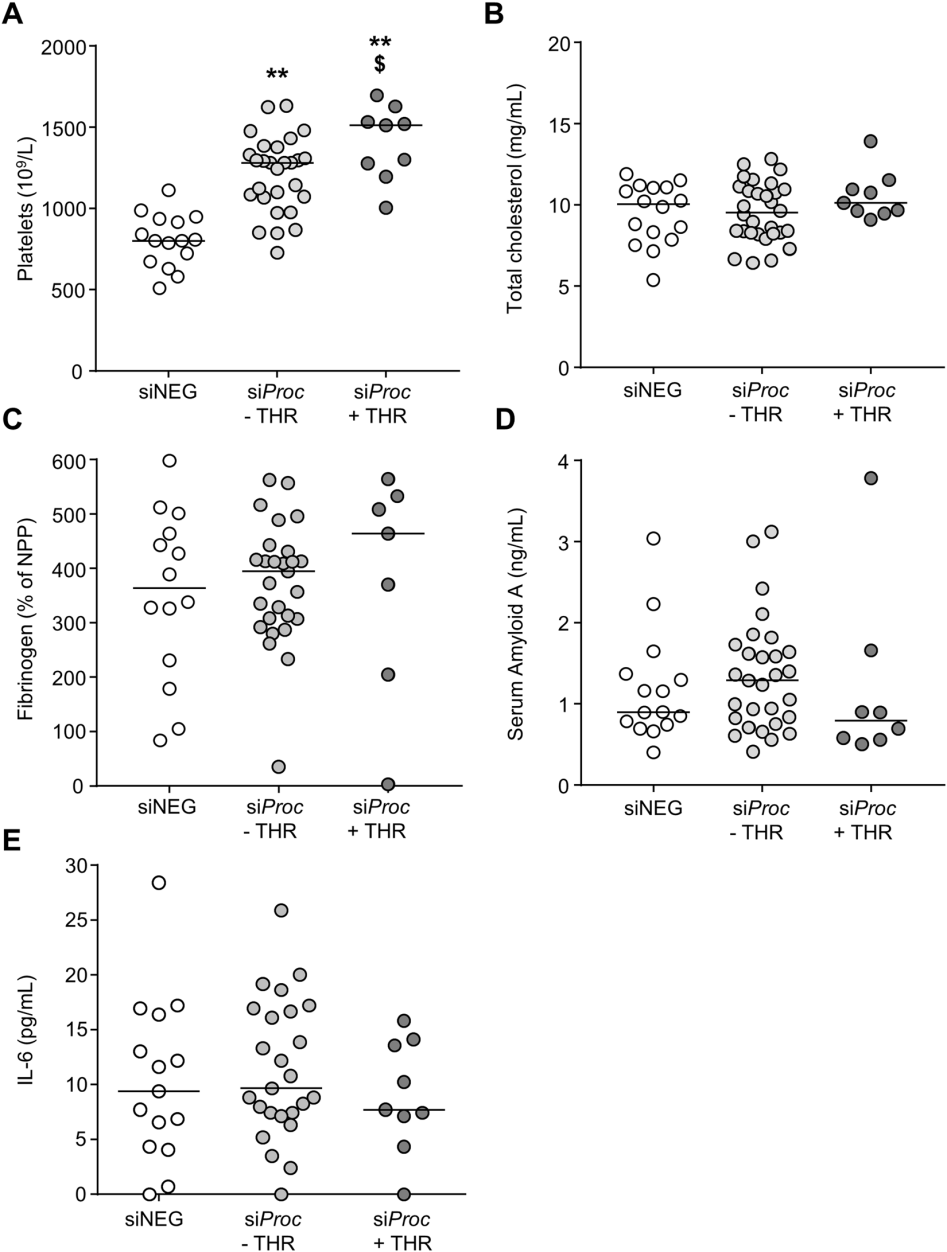
Blood platelet levels and plasma analysis of siNEG and si*Proc* treated *Apoe*^−/−^ mice. (**A**) Total blood platelet levels, (**B**) plasma total cholesterol levels, (**C**) plasma fibrinogen levels, measured by ELISA, and expressed as % of normal pool plasma. We did not have any reason to exclude outliers with a low value (e.g. due to coagulation upon blood withdrawal). (**D**) Serum Amyloid A levels, and (**E**) plasma IL-6 levels (negative measurements were set to 0 pg/mL). Black bars indicate the median. **P < 0.01 for siNEG vs. si*Proc*. ^$^*P* < 0.05 for si*Proc* − THR vs. si*Proc* + THR.

Blood cell counts (total white blood cells, neutrophils, lymphocytes, monocytes, eosinophils, and red blood cells) were comparable between siNEG, si*Proc* − THR, and si*Proc* + THR mice (Fig. 4). Interestingly, platelet levels were significantly higher in si*Proc* treated mice compared to siNEG treated mice (siNEG: 800 × 10^9^/L (508–1112), si*Proc* − THR: 1280 × 10^9^/L (728–1632), si*Proc* + THR: 1512 × 10^9^/L (1004–1696), *P* < 0.001, Fig. 5A). Within the si*Proc* treated groups, si*Proc* + THR mice had even more platelets (*P* = 0.043 as compared to si*Proc* − THR, Fig. 5A). To follow-up on this observation, we determined mRNA levels of megakaryocytes in the spleen of genes associated with platelet production. In agreement with the increased platelet counts, upon si *Proc* treatment,expression of some of these genes (*Gata1*, *Itga2b*, *Nfe2*, and *Fog1*) were increased as compared to siNEG treated mice (data not shown). si*Proc* + THR treated mice showed an increased expression for *Fog1*, while for the other spleen-expressed genes expression levels were not different between si*Proc* − THR and si*Proc* + THR mice.

Plasma analysis showed that total cholesterol levels were not significantly different between mice treated with siNEG or si*Proc*, in both − THR or +THR mice (siNEG: 10.1 mg/mL (5.4–11.9), si*Proc* −THR: 9.5 mg/mL (6.4–12.8), si*Proc* + THR: 10.1 mg/mL (9.1–13.9), *P* = 0.43, Fig. 5B). Besides total cholesterol, fibrinogen, serum amyloid A (SAA, an acute-phase mouse protein associated with inflammation^14^), and interleukin 6 (IL-6, a well-established plasma marker for inflammation (see^15^) were determined as putative systemic markers for atherothrombosis susceptibility. In line with fibrinogen being a cardiovascular risk factor in epidemiological studies^16^, plasma fibrinogen levels in all groups of *Apoe*^−/−^ mice were significantly higher than levels measured in normal pool plasma (NPP, pool of C57BL/6 mouse plasma). However, among siRNA treated *Apoe*^−/−^ mice no differences were observed (siNEG: 363% (83–598), si*Proc* − THR: 394% (35–562), si*Proc* + THR: 463% (2.6–564), *P* = 0.74, Fig. 5C). SAA levels were slightly increased in plasma of the *Apoe*^−/−^ mice compared to NPP (<0.5 ng/mL), but no differences among the *Apoe*^−/−^ groups were found (siNEG: 0.9 ng/mL (0.4–3.0), si*Proc* − THR: 1.3 ng/mL (0.4–3.1), si*Proc* + THR: 0.8 ng/mL (0.5–3.8), *P* = 0.33, Fig. 5D). A similar pattern was observed for IL-6 plasma levels (siNEG: 9.4 pg/mL (0–28.4), si*Proc* − THR: 9.7 pg/mL (0–25.9), si*Proc* + THR: 7.7 pg/mL (0–15.8), *P* = 0.59, Fig. 5E). In line with previous results, PE treatment did not influence blood platelets or plasma levels of the measured parameters (Supplemental Fig. 4).

**Figure 6:**
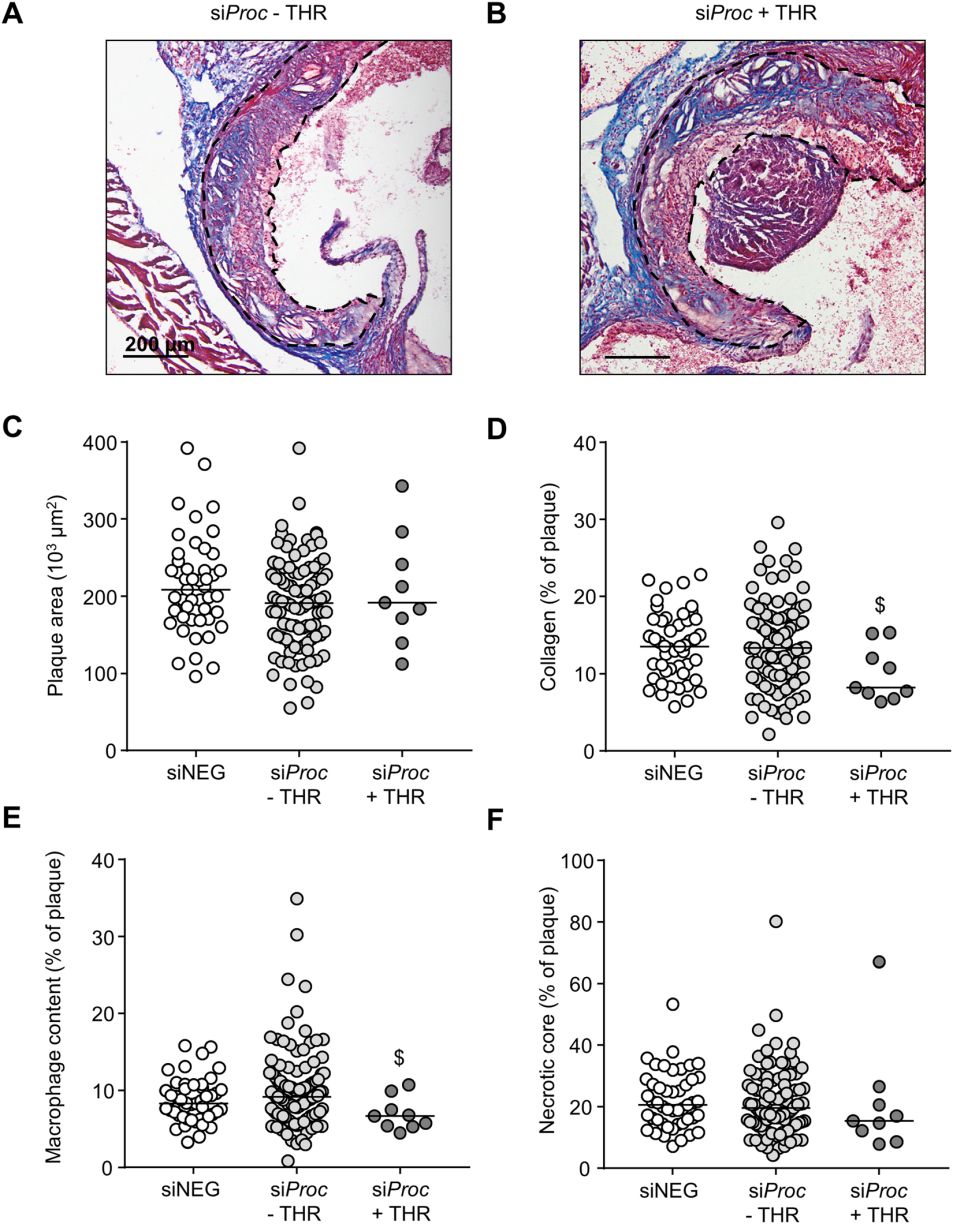
Plaque composition of siNEG and si*Proc* treated *Apoe*^−/−^ mice. (**A**,**B**) Representative photomicrograph of a Masson’s Trichrome stained plaque in the aortic root (RCS) of mice treated with *siProc*, without (**A**) and with (**B**) a thrombus associated to a plaque from the aortic root. Blue areas were assessed as collagen-positive, non-stained areas as necrotic core, and pink nucleated areas as macrophage content/foam cells. The scattered black lines mark borders of the plaque. Black bars represent 200 μm. (**C**–**F**) Individual plaques from the si*Proc* treated group are divided in plaques without a thrombus (si*Proc* − THR) and plaques containing a thrombus (si*Proc* + THR). (**C**) Total plaque area, (**D**) collagen, (**E**) cellular content, (**F**) necrotic core. For all panels, the indicated values represent an average measurement of three sections. Black bars indicate the median. ^$^*P* < 0.05 for si*Proc* − THR vs. si*Proc* + THR.

In our previous study^9^, we found no significant correlation between plaque size and phenotype and the susceptibility to aortic root atherothrombosis. However, with only 4 si*Proc* + THR mice the power in that study was low. Therefore, in the current study the atherosclerotic plaques in the aortic root were also morphometrically analyzed to assess whether plaque size and characteristics could be linked to the presence of atherothrombosis (for examples of plaque stainings, see Fig. 6A,B). Plaque analysis revealed that PE treatment, similar to the atherothrombosis incidence, did not influence any of the plaque characteristics (Supplemental Fig. 5). For this reason, data of the mice with and without PE treatment were pooled to increase statistical power. In the si*Proc* groups, the plaque area (−THR: 191 × 10^3^ μm^2^ (55.0–888), +THR: 191 × 10^3^ μm^2^ (112–343), *P* = 0.59, Fig. 6C), was not different. Interestingly, collagen and macrophage content were significantly lower in plaques with an associated thrombus (−THR: 13.4% (2.1–29.6), +THR: 8.3% (6.4–15.3), *P* = 0.031, Fig. 6D, and − THR: 9.1% (0.8–34.9), +THR: 6.7% (4.5–13.7), *P* = 0.028, Fig. 6E, respectively), unlike the necrotic core (−THR: 19.5% (4.3–80.2), +THR: 15.3% (7.8–67.0), *P* = 0.30, Fig. 6F). No evidence was found for the occurrence of intraplaque hemorrhages or other abnormalities in the atherosclerotic plaques (neither in − THR nor in +THR plaques).

**Figure 7:**
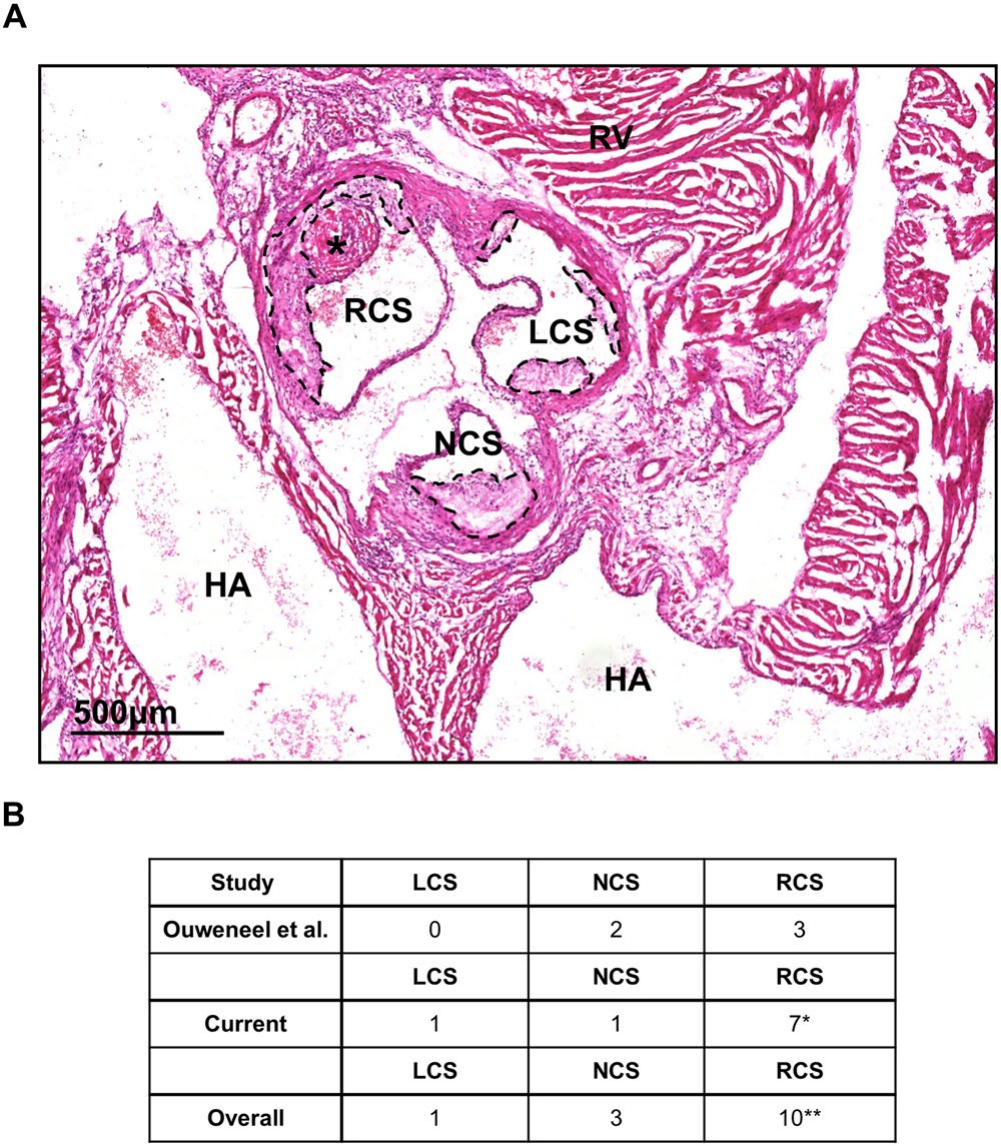
si*Proc* induced atherothrombosis in the different sinuses of the aortic root of *Apoe*^−/−^ mice. (**A**) HE stained section of the aortic root and surrounding tissues. The non-coronary sinus (NCS) of the aortic root is covered by the two atria of the heart (atria are indicated with HA). Left and right of the NCS the left and right coronary sinuses (LCS and RCS, respectively) are present, which are both partly covered by a muscle-rich area, from which the largest part is the right ventricle (indicated with RV). *Thrombus. The scattered black lines mark borders of the plaque. Black bar represents 500 μm. (**B**) Overview of three independent studies, where atherothrombosis in the aortic root occurred upon si*Proc* treatment. *P < 0.05, **P < 0.01.

Since in our studies thrombi were exclusively found in the aortic root, we hypothesized that local hemodynamics, which can influence endothelial integrity, plays a role in atherothrombosis formation. For this reason, we re-examined the individual plaques formed in the different cusps within the aortic root (sinuses) for the presence of thrombi, by dividing the aortic root into the left coronary sinus (LCS), right coronary sinus (RCS), and non-coronary sinus (NCS, see^17–19^ and Fig. 7A). We noticed that thrombi had a preference for the RCS, over the LCS and the NCS (Fig. 7B, *P* = 0.018). When combining these data with the data obtained from our previous study, where we detected 4 thrombi for in total 29 si*Proc* injected *Apoe*^−/−^ mice^9^, the preference for the RCS was even more pronounced (*P* = 0.008).

**Figure 8:**
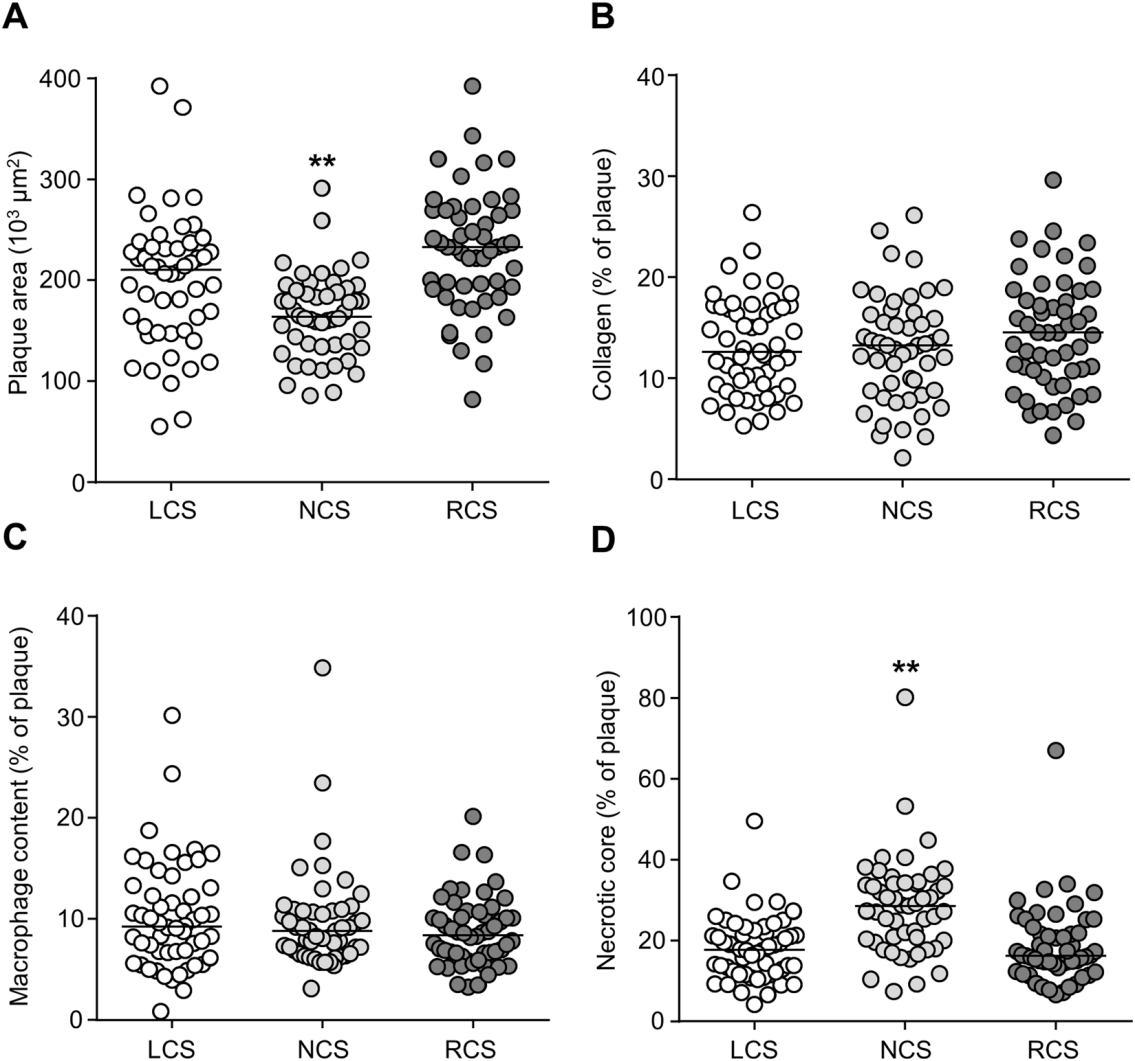
Plaque composition of the LCS, NCS, and LCS. Individual plaques from all groups were pooled and analyzed for (**A**) total plaque area, (**B**) collagen, (**C**) cellular content, (**D**) and necrotic core. For all panels, the indicated values represent an average measurement of three sections. Black bars indicate the median. LCS: Left coronary sinus, NCS: Non-coronary sinus, RCS: Right coronary sinus. **P < 0.01.

To study whether the predilection for the RCS was the consequence of differences in plaque size and composition, the plaque parameters from Fig. 6 were reanalyzed for the LCS, NCS, and RCS. Interestingly, when we pooled the data from the three groups of mice (siNEG, si*Proc* − THR, and si*Proc* + THR) to compare the plaque area, collagen content, macrophage content, and necrotic core size, we found that plaques in the NCS were significantly smaller and contained a larger necrotic core, as compared to their counterparts in the LCS and RCS (Fig. 8). No significant differences among plaques were found when mice were separated in siNEG, si*Proc* − THR, and si*Proc* + THR (Supplemental Fig. 6).

## Discussion

Transgenic mice with an impaired lipoprotein metabolism, such as *Apoe*^−/−^ and *Ldlr*^−/−^ mice, which are fed a cholesterol-rich diet rapidly develop advanced atherosclerosis^1,2^. However, in contrast to humans, these mice only develop atherothrombotic events upon additional (invasive) plaque damaging interventions^20^. The cause for the absence of atherothrombosis is unknown, but it is likely that multiple species-specific factors such as hemodynamics, plaque composition, metabolism, and life span are involved^21^. Recently, our group showed that transient lowering of the natural anticoagulant protein C in *Apoe*^−/−^ mice resulted in spontaneous atherothrombosis at plaques formed in the sinuses of the aortic root^9^. This implies that potent anticoagulation (co)contributes to absence of atherothrombosis in mice. In the current study we aimed to increase the insight into the factors which associate with and possibly determine when and where a thrombus develops on top of an atherosclerotic plaque. For the first time we provide evidence that atherosclerotic plaques located in the RCS of the aortic root are more prone to the development of atherothrombosis, compared to the LCS and NCS. Interestingly, plaques with a thrombus were modestly but significantly lower in collagen and macrophage content. Therefore, we conclude that low protein C-induced atherothrombosis in mice is a low incident but reproducible phenomenon, and that it is determined by the location of the plaque, possibly in combination with differences in plaque composition.

Since Leonardo da Vinci in the beginning of the XVI century, scientists have been intrigued by the sophisticated and sustainable tissue and milieu of the aortic valves and root^22–24^. The mouse aortic sinuses (in humans, sinuses of Valsalva) play a crucial role in regulating the closing mechanism of the valve leaflets^25^. The oscillating shear stress due to the opening and closing of the valves renders the cusps susceptible for the development of atherosclerotic plaques. A limited number of studies have distinguished between the three different sinuses and their plaques during their analyses. It has been reported that plaque size is reduced specifically in the RCS upon treatment with two different liver X receptor agonists^18^. This means that differences in local anatomy of the aortic root do not only cause the flow to be different, but also can induce different expression patterns of proteins, possibly linked to coagulation. Moreover, Bentzon *et al*. suggested a causal relation between lesion formation and expansive remodeling of the aortic root sinuses, which was different when comparing plaques in the LCS, NCS, and RCS^26^. In our study, we found that plaques formed in the RCS, the location where most thrombi are identified, and the LCS are significantly larger than their counterparts NCS. In addition, plaques in the NCS have a larger necrotic core as compared to the LCS and RCS. This observation again suggests that the plaques in the different sinuses are not similar, but since no outlying characteristics were scored for the RCS we cannot speculate on whether plaque composition contributes to atherothrombosis. The observed preference for the RCS for the development of atherothrombosis suggests that in mice local hemodynamics and wall shear stress are not only involved in atherogenesis^27,28^, but also in the development of atherothrombosis, as has been proposed for the human disease^29,30^. The identification of factors that drive the predilection for the RCS may provide novel clues on factors that drive atherothrombosis in mice and humans.

Plaques in the aortic root which were associated with a thrombus appeared to be lower in macrophage and collagen content. Although the measured values fell within the normal range, the difference was considered statistically different. Macrophages (foam cells) are crucially involved in atherogenesis and a high content of this cell population within a plaque has been associated with instability^31,32^. These observations make our findings in the current study, where in atherothrombotic plaques relatively fewer macrophages were found, unexpected. However, it should be noted that the plaques were advanced and that as a result the macrophage foam cell content was low. It has been described that lower collagen levels in atherosclerotic plaques are associated with instability and risk of rupture^33^. However, because of the current study setup, we cannot conclude whether the observed difference is a cause or consequence of the formation of a thrombus.

In the current study, si*Proc* treatment of *Apoe*^−/−^ mice and subsequent thrombus formation did not coincide with altered levels of red and white blood cells or plasma markers of inflammation (fibrinogen, SAA, and IL-6). The only blood cell population which was altered upon si*Proc* treatment were the platelets. Most likely due to increased platelet production, atherosclerotic si*Proc* treated mice showed significant higher levels of platelets in the circulation. To measure platelet production, a transcript analysis for genes associated with platelet production was performed in the spleen. The spleen does not contribute equally to production of platelets in mice, as compared to the bone marrow^34^. However, the spleen does contain megakaryocytes, which derive from hematopoietic stem cells^35^. In addition, mice deficient for the *Apoe* gene have increased extramedullary hematopoiesis^36^, which means that the contribution of the spleen to total platelet production is increased in these animals. Measuring spleen megakaryocytes to detect alterations of platelet production upon si*Proc* treatment is thus a valid alternative for the bone marrow in *Apoe*^−/−^ mice. Indeed we measured an upregulation of genes associated with platelet production. In addition, a follow-up study showed that in the absence of an atherosclerotic phenotype (in an independent experiment, in which wild type female C57BL/6 mice were fed a normal chow diet) platelet numbers were also increased in mice treated with an siRNA against *Proc* (data not shown). Both findings suggest that the anticoagulant protein C directly or indirectly influences platelet production (and possibly consumption and/or clearance), a phenomenon that, to our knowledge, has not been described before. In human studies, it has been suggested that increased platelet levels are a risk factor for atherothrombotic events, making it tempting to speculate that the higher platelet levels upon si*Proc*-treatment contribute to the development of atherothrombosis in the aortic root^37,38^. For now, it is however not clear whether the increased platelets predict aortic root atherothrombosis susceptibility or whether they are a consequence of the event. For future studies, it would be of interest to dig deeper into the role of platelets and platelet activation during si*Proc*-mediated atherothrombosis in the aortic root. For instance, modulation of platelet function (e.g. via GPVI, see^39^) in combination with protein C silencing may coincide with a different onset and progression of this unique atherothrombotic phenotype in mice. Moreover, the role of platelets during the local event of atherothrombosis can be studied in intravital microscopy experiments with fluorescently labeled platelets. However, because it is technically not possible to perform these type of studies in the aortic root, this is currently not feasible.

In conclusion, atherosclerotic plaques in the RCS are a predilection site for low protein C-induced spontaneous atherothrombosis in *Apoe*^−/−^ mice, a process associated with vulnerable plaques displaying a lower macrophage and collagen content and vulnerable blood with augmented platelet counts.

## Materials and Methods

### Animals

Female C57BL/6 *Apoe*^−/−^ mice (4–7 weeks old, n = 57) were fed a Western-type diet (WTD containing 0.15% cholesterol; Special Diet Services, Sussex, UK). After 6 weeks on WTD, mice received silicone collars around both common carotid arteries, as previously described^11^. After 10 weeks on WTD, synthetic siRNA (5 mg/kg; Ambion, Life Technologies, Carlsbad (CA), USA) targeting protein C (si*Proc*, cat. #S72192; n = 40), or a control siRNA without a known target in mice (siNEG, cat. #4404020; n = 17) was injected intravenously (IV). siRNA was complexed using invivofectamine 3.0, and each mouse received 2.5 nmol siRNA. Prior to siRNA injection, mouse groups were randomized based on weight and age (Table 1). siNEG- and si*Proc* treated mice received two intravenous injections of either phenylephrine (PE, 8 μg/kg IV, Sigma-Aldrich cat. #P6126–10G, Zwijndrecht, The Netherlands; si*Proc*: n = 20, siNEG: n = 8) or Phosphate-Buffered Saline (PBS; si*Proc*: n = 20, siNEG: n = 9) as control. PE/PBS injections were performed 4 and 6 days after the siRNA injection. In both the siNEG and the si*Proc* group, one mouse was removed from the experiment due to procedural errors, and thus not represented in the analyses. For an overview of the experimental procedure, see Supplementary Fig. 7. The animal experimental protocol was in agreement with the national guidelines for animal experimentation and was approved by the Ethics Committee for Animal Experiments (Leiden University, Leiden, The Netherlands).

**Table 1:** Group sizes, age, and body weight for all *Apoe*^−/−^ mice.

Group	Group size (n)	Age (weeks)	Body weight (g)
siNEG	17	14 (13–16)	22.5 (20.9–27.9)
si*Proc*	40	14 (13–16)	22.7 (20.1–25.7)
siNEG + PBS	9	14 (13–16)	22.4 (20.9–27.9)
siNEG + PE	8	15 (13–15)	22.9 (22.1–24.2)
si*Proc* + PBS	20	14 (13–16)	22.6 (20.3–24.9)
si*Proc* + PE	20	14 (13–16)	22.8 (20.1–25.7)

For age and body weight, the median and range is displayed of both parameters before siRNA injection. There were no significant differences between groups of mice.

### Tissue harvesting and preparation

7 days after siRNA injection, mice were anesthetized with a subcutaneous injection of a mixture of ketamine (100 mg/kg), xylazine (12.5 mg/kg) and atropine (125 µg/kg). Citrated blood (300 μL) was drawn from the inferior vena cava, and plasma was collected as described previously^40^. For whole blood cell and platelet analysis, an EDTA blood sample (approximately 50 μL) was collected through retro-orbital bleeding after blood withdrawal from the vena cava. Subsequently, mice were further exsanguinated and perfused *in situ* with PBS, after which organs were harvested. Tissues were either snap frozen (liver and spleen, stored at −80 °C), or fixed for 24 hours in 3.7% neutral-buffered formalin (heart and carotid arteries, Formal-Fixx, Shandon Scientific Ltd, UK) and stored at room temperature for analysis.

### Gene expression evaluation

Transcript of *Proc* was assessed by routinely quantitative PCR of hepatic tissue, with *Actb* as a reference housekeeping gene^41^.

### Histology

Hearts (aortic roots) and carotid arteries were formalin fixed and embedded in OCT compound (Optimum Cutting Temperature; Sakura Finetek Europe B.V., Alphen aan de Rijn, The Netherlands) before sectioning. Cryosections (10 μm, 70 μm interval) of the aortic root were prepared and mounted in a parallel series on 1% gelatin coated slides. Thrombi and clot structures in the aortic root were identified and scored blindly by two independent researchers on hematoxylin and eosin-stained sections. Plaque size per valve was determined after staining of the cyrosections for neutral lipids using Oil-Red-O and hematoxylin (Sigma-Aldrich, Zwijndrecht, The Netherlands). Corresponding sections were stained using the Masson’s Trichrome method (Sigma-Aldrich) for further morphometric analysis of the atherosclerotic plaques, including the percentage of collagen, necrotic core, and macrophage foam cells. All images were analyzed by blinded computer aided morphometric analysis using the Leica DM-RE microscope and LeicaQwin software (Leica Ltd, Cambridge, UK). Transverse serial cryosections (10 μm; 80 μm interval) of the carotid arteries (and the collars) were made. Sections were routinely stained with hematoxylin and eosin for a general assessment of histology, and were analyzed blindly by two independent researchers.

### Blood and plasma analysis

Whole blood cell and platelet analysis were performed using an automated XT-2000iV veterinary hematology analyzer (Sysmex Europe GMBH, Norderstedt, Germany). Plasma cholesterol levels were measured by an enzymatic colorimetric assay (Roche diagnostics, Almere, The Netherlands). Protein C plasma levels were determined using an ELISA with a sheep anti-murine protein C polyclonal antibody (Haematologic Technologies Inc. Essex Junction (VT), USA), conjugated with Horseradish peroxidase with the EZ-Link Plus Activated Peroxidase Kit (Thermo Scientific, #31489, Waltham (MA), USA), as described previously^41^. Fibrinogen and Serum Amyloid A plasma levels were determined using commercial ELISA kits (Affinity Biologicals, Ancaster (ON), Canada and R&D systems, Minneapolis (MN), USA, respectively). Plasma IL-6 levels were determined using a commercially available ELISA kit (BD Biosciences, San Jose CA, USA) following the manufactures instructions.

### Statistics

Statistical analyses were performed in Graphpad Instat (GraphPad Software, La Jolla (CA), USA). Data are presented with median and range. Statistical differences were assessed using the Mann-Whitney U test. A *P*-value < 0.05 was considered significant. Descriptive statistic and calculate proportions of the observations and the 95% confidence intervals (Wilson-score) were calculated using resources provided by the Open Source Epidemiologic Statistics for Public Health website (www.openepi.com). A Chi-Square test was performed for the valve-incidence was done in SPSS (IBM SPSS Statistics for Windows, Version 23.0, Armonk (NY), USA).

## Electronic supplementary material

Supplementary figures
