# Supplementary material for: Predilection of Low Protein C-induced Spontaneous Atherothrombosis for the Right Coronary Sinus in Apolipoprotein E deficient mice
Source: Sci Rep. 2018 Oct 10;8:15106. doi: 10.1038/s41598-018-32584-y (PMC6180072; doi:10.1038/s41598-018-32584-y)
Supplement: Supplementary file 1 — Supplementary figures [file 41598_2018_32584_MOESM1_ESM.pdf]

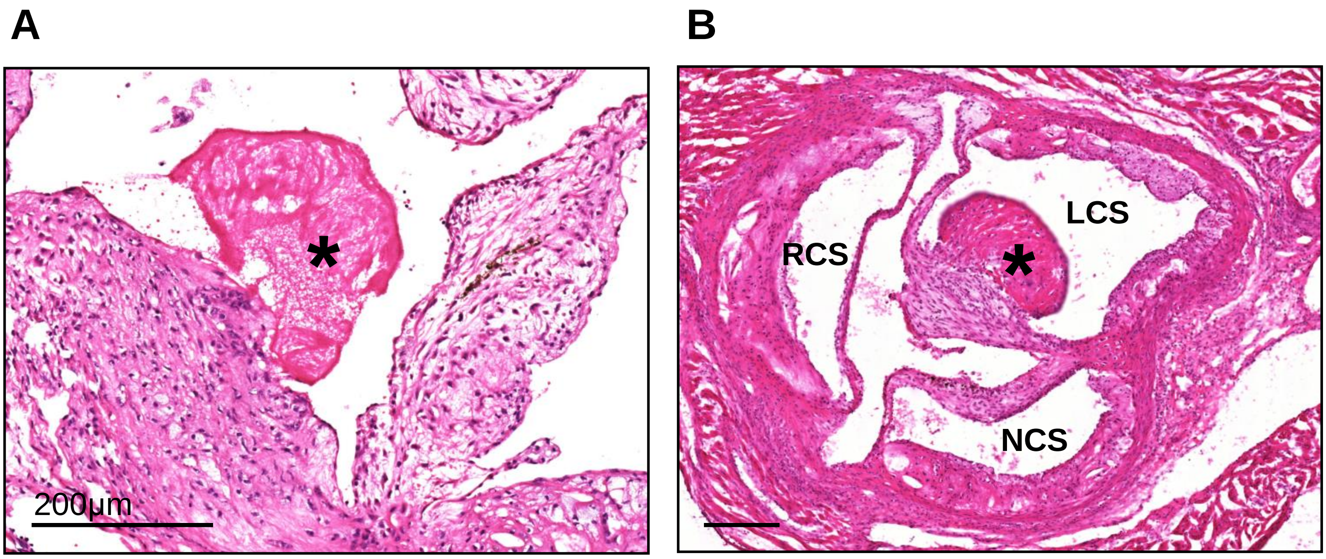

Supplemental figure 1: Atypical thrombi in the aortic root of si*Proc* treated *Apoe*$^{-/-}$ mice. (A) Atypical thrombus which associated with an atherosclerotic plaque. In contrast to other thrombi, it did not have a layered structure and did not contain leukocytes. (B) Atypical thrombus associated with a valve within a sinus of the aortic root, and not with the atherosclerotic plaque. The composition of the thrombus was similar to other thrombi. During the analysis, both mice were included in the si*Proc* –THR group. Black bars represent 200 µm. NCS: Non-coronary sinus, RCS: Right coronary sinus, LCS: Left coronary sinus. *: Thrombus.

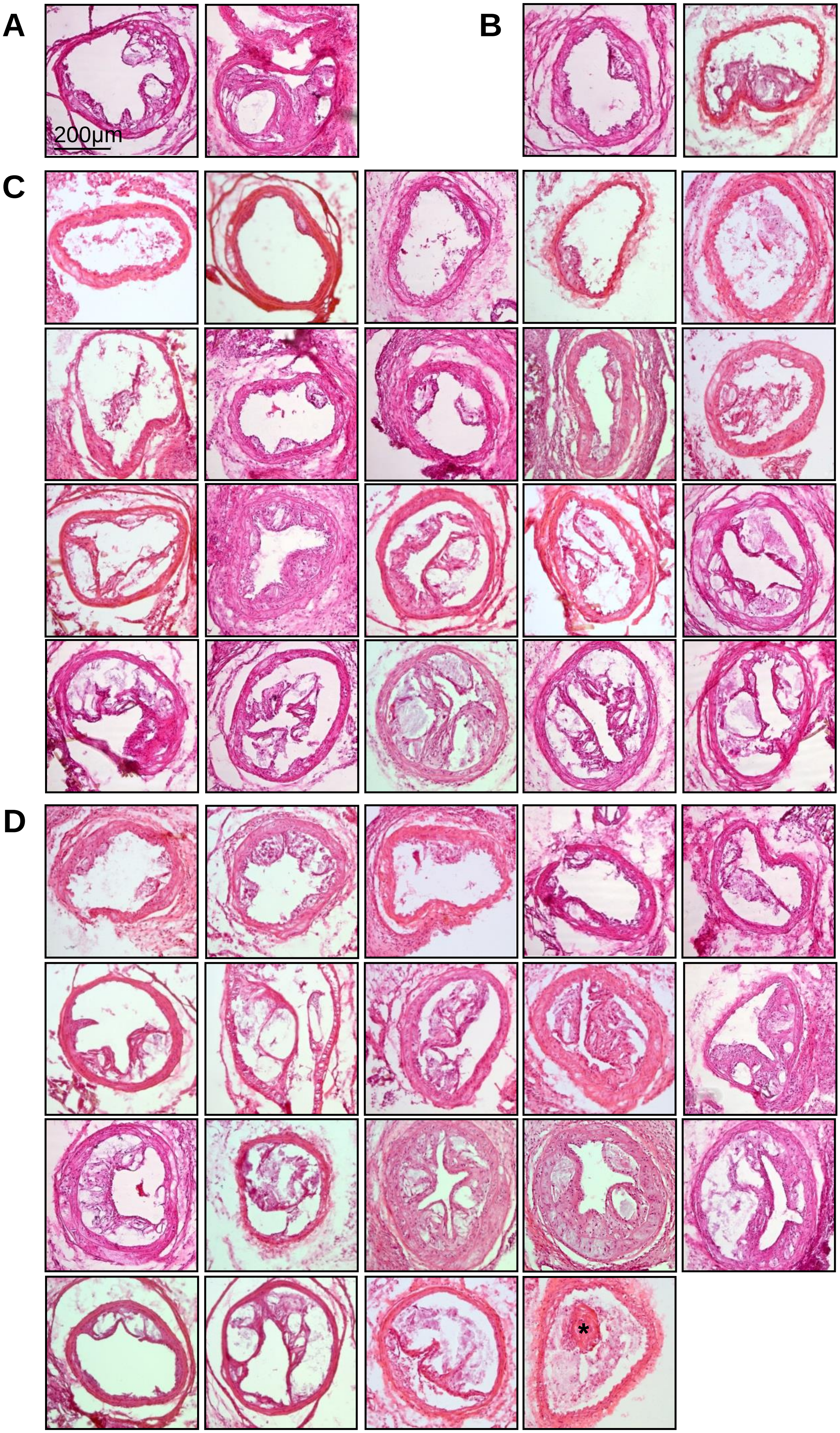
A
B
200μm
C
D
*

Supplemental figure 2: Atherosclerotic plaques upon collar placement in the common carotid arteries. Overview of sectioned common carotid arteries, at the site of maximal stenosis. (A) Two representative sections of common carotid arteries of mice treated with siNEG (- PE), (B) Two representative sections of common carotid arteries of mice treated with siNEG (+ PE), (C) Sections of common carotid arteries of mice treated with si*Proc* (- PE), (D) Sections of common carotid arteries of mice treated with si*Proc* (+ PE). All sections were HE stained. Black bar represents 200 µm. *: Thrombus (for enlargement, see figure 1F).

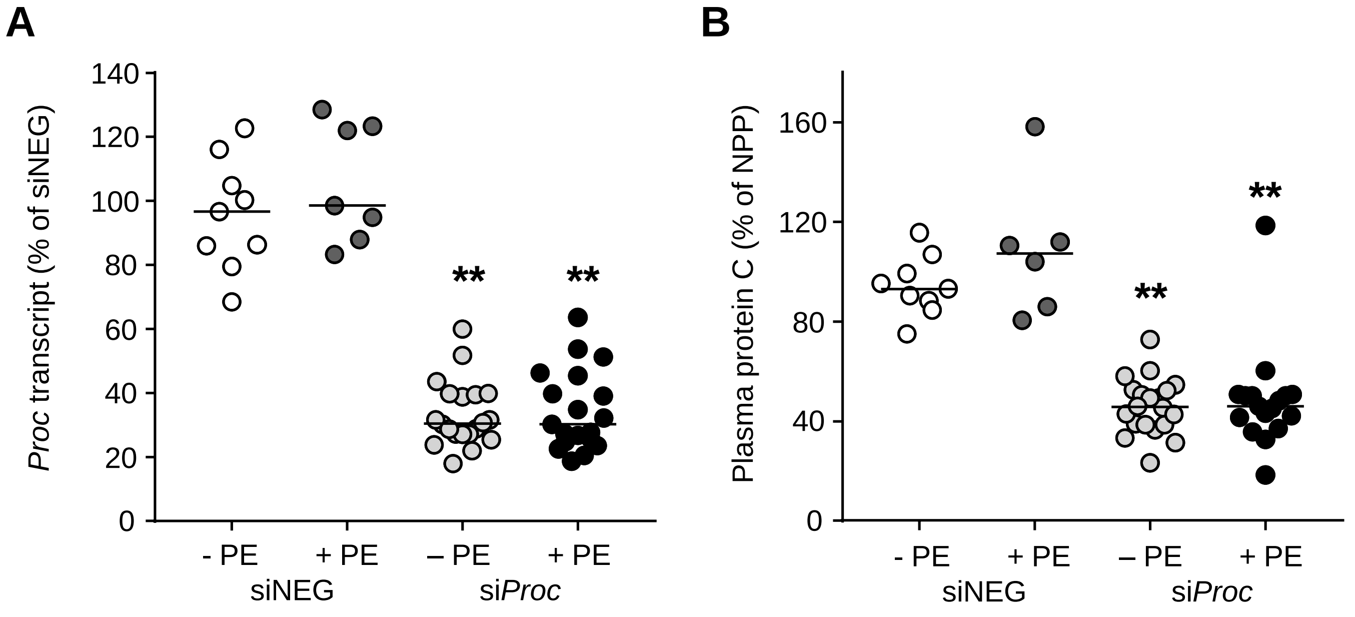

Supplemental figure 3: *Proc* liver transcript and plasma protein C levels in siNEG and si*Proc* treated *Apoe*$^{-/-}$ mice, with and without phenylephrine (PE). (A) *Proc* transcript in the liver upon sacrifice (7 days after si*Proc* treatment), compared to the mean value of siNEG treated (100%) and (B) plasma protein C levels, compared to the mean value of siNEG treated (100%). Black bars indicate the median. – PE: PBS control treated mice, + PE: Phenylephrine treated mice **: *P*<0.01 for siNEG vs. *siProc*.

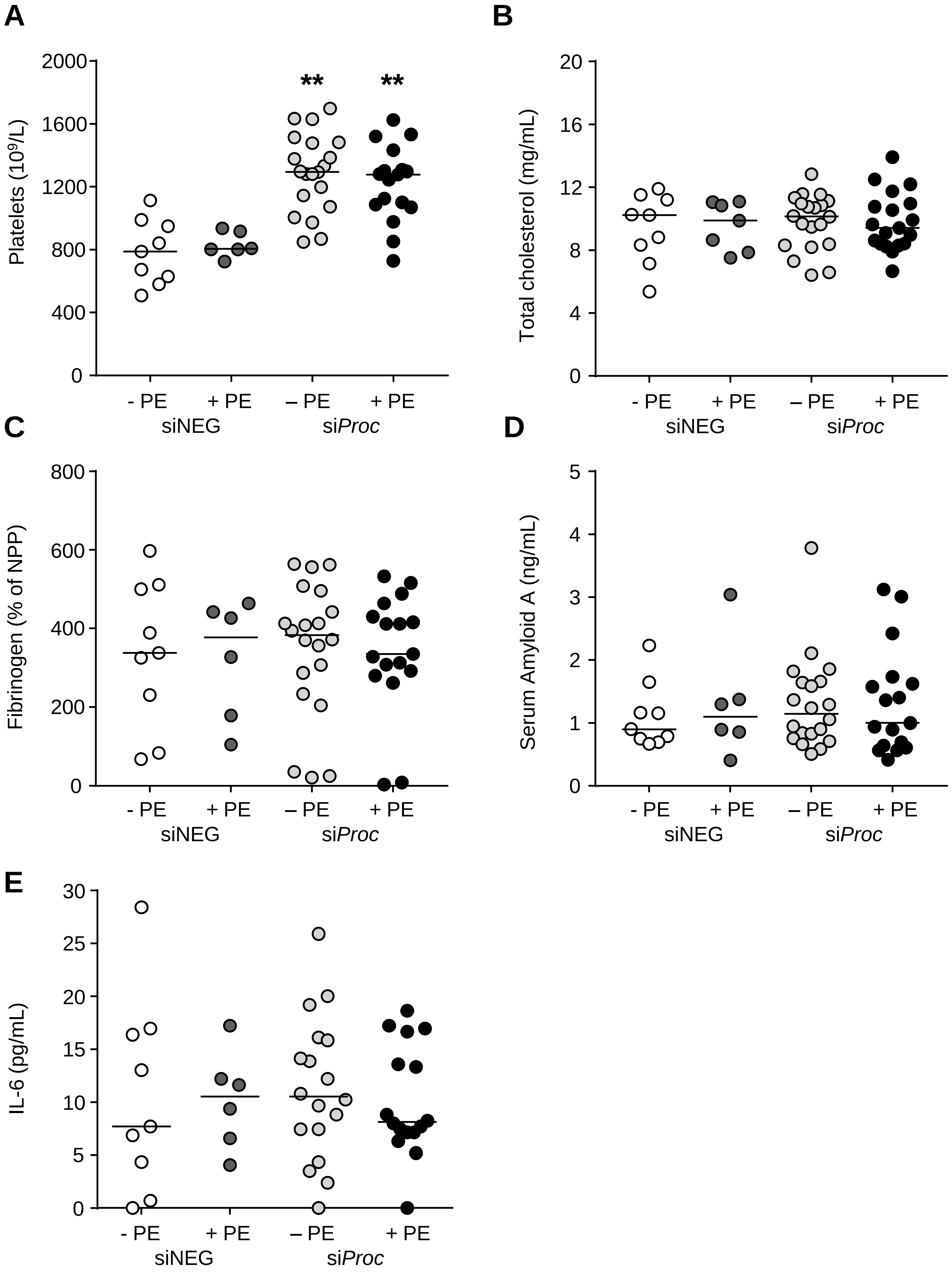

A
Platelets (10⁹/L)
2000
1600
1200
800
400
0
**
**
- PE
+ PE
– PE
+ PE
siNEG
siProc
B
Total cholesterol (mg/mL)
20
16
12
8
4
0
C
Fibrinogen (% of NPP)
800
600
400
200
0
D
Serum Amyloid A (ng/mL)
5
4
3
2
1
0
E
IL-6 (pg/mL)
30
25
20
15
10
5
0

Supplemental figure 4: Blood platelets and plasma markers of siNEG and si*Proc* treated *Apoe*$^{-/-}$ mice, with and without phenylephrine (PE). (A) Whole blood platelet levels, (B) plasma total cholesterol levels, (C) plasma fibrinogen levels, measured by ELISA and expressed as % of normal pool plasma. We did not have any reason to exclude outliers with a low value (e.g. due to coagulation upon blood withdrawal), (D) Serum Amyloid A levels, and (E) plasma IL-6 levels (negative measurements were set to 0 pg/mL). Black bars indicate the median. – PE: PBS control treated mice, + PE: Phenylephrine treated mice **: P<0.01 for siNEG vs. *siProc*.

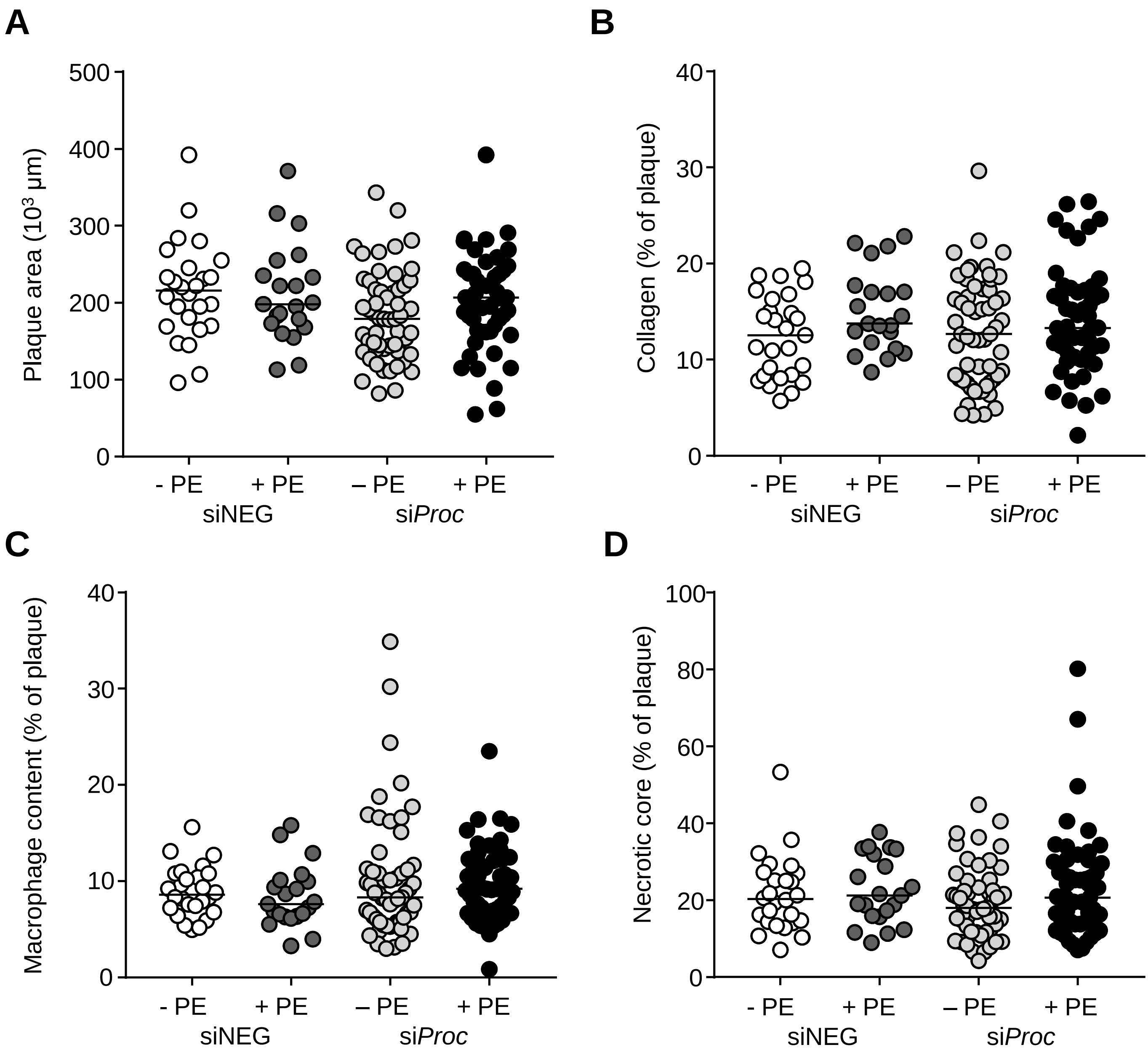

Supplemental figure 5: Atherosclerotic plaque composition of siNEG and si*Proc* treated *Apoe*$^{-/-}$ mice, with and without phenylephrine (PE). (A) Total plaque area, (B) collagen, (C) macrophage content, (D) necrotic core. For all panels, the indicated values represent an average measurement of three sections. Black bars indicate the median.

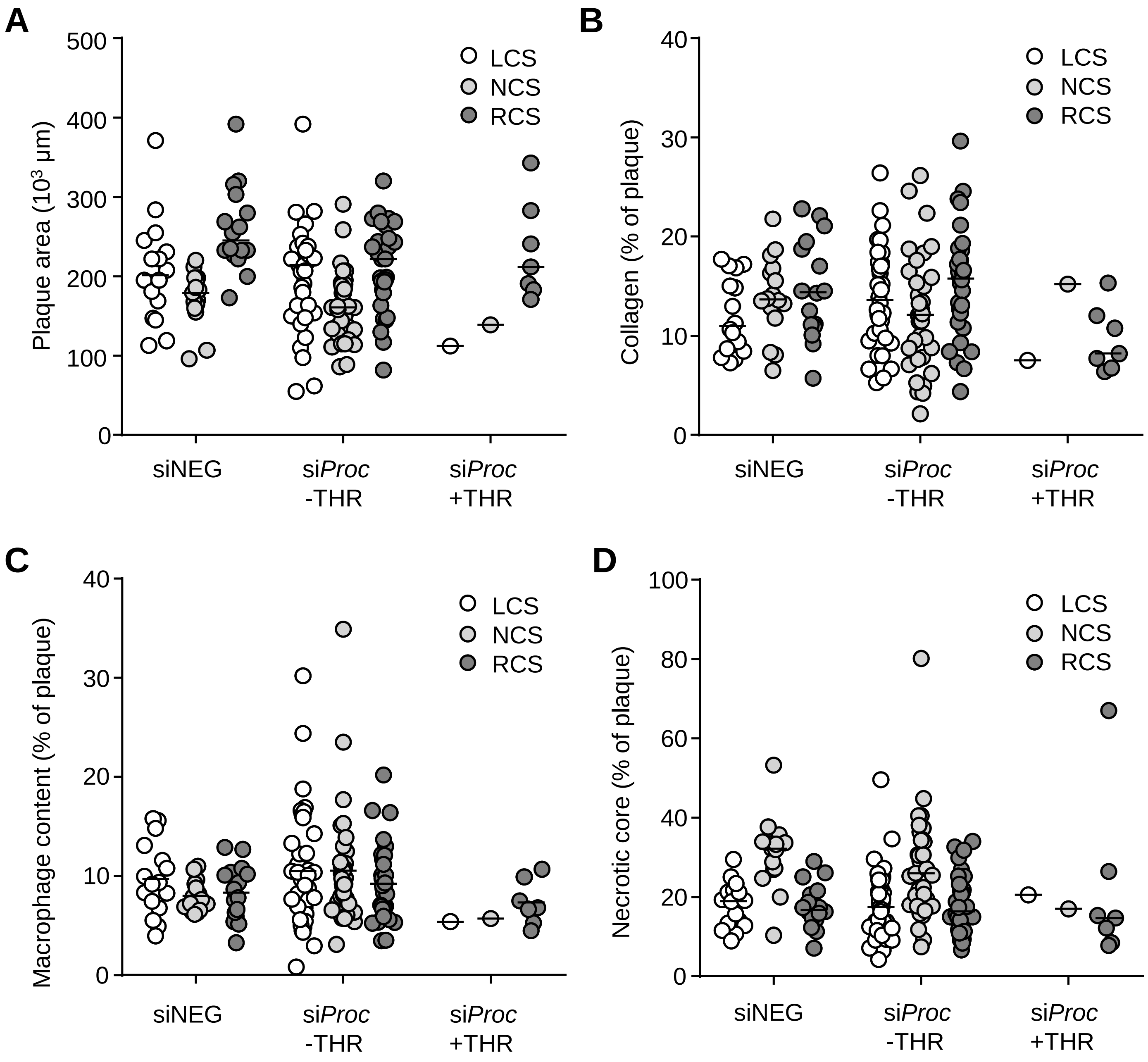

Supplemental figure 6: Composition of atherosclerotic plaques formed in the LCS, NCS, and RCS. (A) Total plaque area, (B) collagen, (C) macrophage content, (D) necrotic core. LCS: Left coronary sinus, NCS: Non-coronary sinus, RCS: Right-coronary sinus. For all panels, the indicated values represent an average measurement of three sections. Black bars indicate the median.

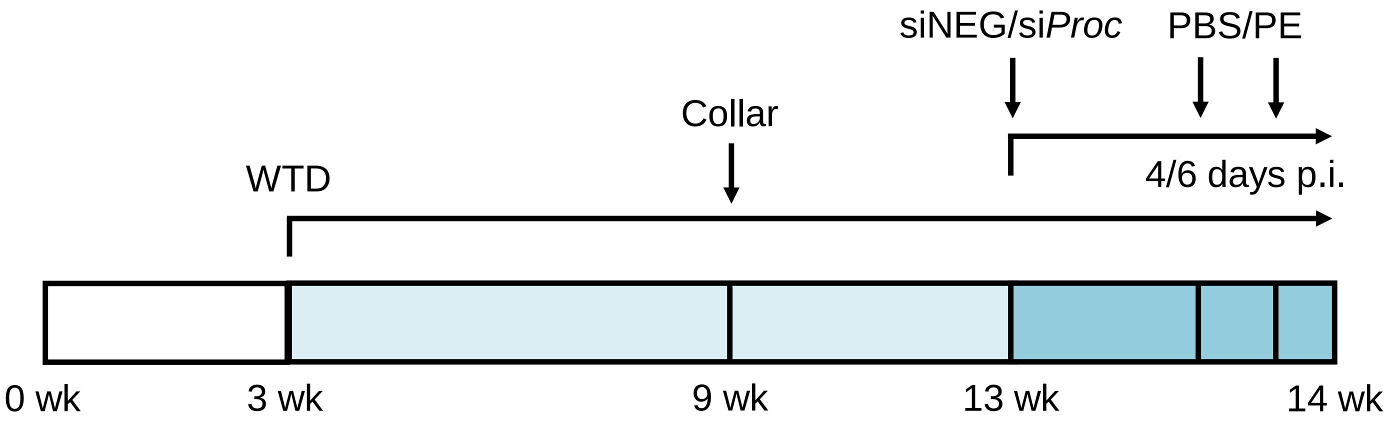

Supplementary figure 7: Experimental setup. 4-7 weeks old female *Apoe*$^{-/-}$ mice were fed a Western type diet (WTD) for 10 weeks (wk). After 6 weeks of WTD, perivascular collars were placed around both the common carotid arteries. After 10 weeks of WTD, mice were treated with either siNEG or si*Proc*. 4 and 6 days after siRNA treatment (post-injection, p.i.), mice were injected with PBS control or phenylephrine (PE). One week after siRNA treatment (age: 15-18 weeks), mice were sacrificed.
